# Clinical Lectures on Surgery, Delivered at St. George’s Hospital

**Published:** 1846-08

**Authors:** 


*Clinical Lectures on Surgery, delivered at St. George's Hospital, by* Sir Benjamin C. Brodie, V. P. R. S., *Serjeant-Surgeon to the Queen; Surgeon in ordinary to his royal Highness Prince Albert; etc. etc. etc. Published by Lea & Blanchard, Philadelphia,* 1 *vol. 8vo.* 1846.

Sir Benjamin Brodie, Serjeant-Surgeon to the Queen of England, which highest surgical honor he held during the two preceding reigns, has a plain, open, but thoughtful face, and wears his coat loosely, and all this you may see in every page of the book before us. The lectures were delivered at various periods before the students of this princely charity, and embody the results of his experience both there and in an extensive private practice during a tolerably long life. They are therefore, what all who know the man would have expected, eminently practical and plain, with no pompous parade of words without ideas, no senseless, wire-drawn speculations, no display of personal success to the prejudice of his weaker brethren and truth — no loud, positive assertion where others find room to doubt, as if to say "I am Sir Oracle." Sir Benjamin has lately resigned his place in St. George's Hospital, because he thought he had occupied it long enough, and he felt bound to give way for others who were laboring for distinction. Already a universal favorite in London, this was the crowning act to his reputation and the profession have since given him signal testimony of their estimate of the generous act. His ability as a surgeon may be none the greater for all this, but certainly it is pleasant to know that our author is a man in whose benevolence and honesty we may implicitly rely. Read his affectionate and familiar address to his pupils on the opening of his annual course, and in the following single paragraph you may learn to appreciate the man.

"But I must say that I have never known any one to do any real good in the world, or obtain ultimately a bright reputation for himself, who did not begin life with a certain portion of humility. The greatest men are humble. Humility leads to the highest distinction, for it leads to self-improvement."

The second lecture treats of "some of the important circumstances connected with operative surgery"— and as we ran along the pages we were attracted to the following paragraphs.

"The surgeon who is engaged in operations must attend in all respects to the mode of his life."

"An acomplished operator! * * * I apply it not to him who looks at his watch to see in how short a space of time an operation may be completed."

Yet the reader has not forgotten how much eclat was given to the name of Dr. ——, of ——, who whittled off a poor fellows thigh in 15 seconds; and the question was never perhaps asked, whether any necessity for the operation actually existed, or whether the operation was not so badly done that a second amputation became necessary? In surgery we hold to the maxim that "what is well done, is quickly enough done," and that whoever cuts against time will make a more humane executioner than surgeon.

Never underrate the dangers of an operation to a patient or to his friends.

"The friends should never have the opportunity of turning round upon him afterwards and saying "you said there was no danger and here my wife, my husband, or my friend, is dead." * * * "If I am asked whether there be any danger, I never answer that there is none." * * "It will be a great comfort and afford much peace of mind in the arduous profession in which you are engaged, if you attend to this advice." * *

"Some people seem to have a notion that the loss of blood in an operation will make the patient less liable to inflammation afterwards. But I believe that it is just the reverse. Bleeding may relieve phlegmonous inflammation where it already exists, but it does not prevent its existence; and on the other hand, I have no doubt that it increases the liability of the patient to other kinds of inflammation, such as erysipelas, or diffuse inflammation of the cellular membrane or venous and artereal inflammation." * * * I was educated in the belief that the thing to be most apprehended after an operation, was some kind of inflammation; and that the way to prevent inflammation was to keep the patient on low diet, and as long as I acted in accordance with these views, I was meeting with erysipelas at every turn of my practice." etc.

What will our leeches say to this, who are forever crying "give, give"? who see no safety while one drop of blood purples the cheek, and who have never ceased to echo the words of Broussais as an apology for their ultra depletent notions: "It is of an inflammation of one or more of the organs essential to life, of which sooner or later a great proportion of the human family perish." Here uprises a heterodoxy so frightfully absurd and dangerous, that the disciples of Rush, at its presence, must start and tremble with holy horror—*excessive depletion, not only does not prevent, but actually causes inflammation*! Yet it is no "*revasseries* or dream of the empty brain," but a real tangible fact which all may see who are not sand blind, nay, gravel blind.

"There is another point, which it is always worth your while to consider before an operation. Has the patient any particular disposition to hemorrhage?"

"Even in the case of a young woman who is more than commonly hysterical, I advise you to proceed with caution. Her powers of life are weak; she will ill-bear any considerable hemorrhage: and she is more liable than others, not only to a dangerous disturbance of the nervous system, but also to those low, inflammatory affections consequent on operations, of which I have spoken formerly." 49.

Following several successive lectures on the important subject of "Mortification" is a lecture or two on "Diseases of Veins."

"Why is it that superfical veins enlarge and not the others? Because the deepseated veins have support on all sides, while the superficial have not, The first thing you have to do then is to support by equal pressure these superficial veins. Where there are only two or three varicose clusters, of small size, you need only apply strips of adhesive plaster. Determine precisely where they are, then elevate the limb so as to empty the veins and apply the plasters in strips of about an inch and a half wide by four inches long, drawing up the skin under them and taking care that the plaster is not thrown into tucks or folds. In a great many cases you will find that this is sufficient to give all the support required, and perhaps this is all that the patient needs for the whole of his life. But when the veins of the leg are extensively varicose this will not be sufficient—you may use here a roller of coarse unbleached calico or of flannel which the patient can apply for himself better than the roller of calico, or of stocking web which is a very nice bandage, but it is expensive and is good for nothing after it has been three or four times washed. Patients who are awkward in applying a bandage may manage the laced stockings, which are made of various materials. *Nanquin* is very good; some are now made partly of India rubber. An ingenious artist in Jermyn street makes a laced stocking of very fine spiral wire included within folds of leather or something else. Each kind has its advantages in particular cases." 118.

In connection with an operation for the obliteration of the vena saphena which terminated fatally, Sir Benjamin remarks:

"Since then, as you may suppose, no operation has been performed on the vena saphena, either by ligature or in any other way. There are no circumstances here to justify the performance of a dangerous operation. You may perform dangerous operations to get rid of a disease still more dangerous, but you have no right to perform an operation attended with such a degree of danger as can be appreciated, in order to get rid of a disease which is not dangerous; and no one can say that varicose veins belong to the class of dangerous diseases. But still there is another reason against having recourse to this operation. I do not believe, from any thing that I have formerly seen, that the operation permanently benefitted the patients."

"The operation, however, is proper where there is a varicose cluster much distended, and liable to burst and bleed."

Among the thousand and one modes suggested and practiced for the radical cure of varicocele, one thing must have been constantly noticed; the authors have each successively declared every other method dangerous, but their own. From Artius of the fifth century down to Davats, Bonnet, Velpeau, Breschet, Fricke, Delpech, Warren, and a host of others in the nineteenth century, the caustic, the ligature and compression have found in one form or another their advocates and assailants. There is a balm, but whether in Physick or Velpeau, Davats or Dupuytren is yet in dispute. Brodie has also a method of his own, yet with singular and becoming honesty he declares its hazard. And of *all* the methods we are ready to employ the language applied by Mr. Liston to that of Beclard, alone: they are "too often followed, and that very speedily, by a *permanent* arrest of the whole circulation, to be persevered in," or as we have said on another occasion "we hold that veins cannot be permanently closed without inflammation, and that all modes of occlusion, whether by ligature, simple or modified, acupuncturation, seton, excision, compression or caustic, are alike liable in certain constitutions, and under certain circumstances, to fatal phlebitis."

The chapter on Corns and Bunions will not fail to interest a large portion of readers, and especially those who being afflicted with a hereditary pride and foppery, inherit also, as suitable armorial bearings, the calamity of small shoes and *corns,* which latter some writer has gravely assured us are constitutional and unavoidable in certain families. Sir Benjamin recommends *first*, a piece of linen spread with diachylon plaster, *or a piece of thick buckskin with a hole in it. Second*, apply nitrate of silver, or nitric acid. *Third*, scrape its surface with a very fine steel, or a fish-skin rasp. *Fourth*, cut it out. *Fifth*, shape the shoe to the foot, or let it be made of a soft and yielding material. *Sixth*, restore the toes, if displaced, to their natural position.

Soft Polypi of the nose should generally be removed by forceps. They are almost always attached to the cells of the ethmoid bone, though occasionally to the superior turbinated bone. p.135. * * * "Now the polypus being removed, the patient will naturally enquire whether it will return, and you must answer that most likely it will. But then he will probably put another question. Can you do any thing to prevent its recurrence, or to make its growth slow? I believe that you may retard its return in many cases, or even prevent it. You may for this purpose apply daily with a camel's hair pencil to the part from which the polypus originated, the white precipitate ointment, softened a little by holding it to the fire; and it must be persevered in not for a few days, a few weeks, or a few months, but even for years. The application must be thorough or it can do no good." p. 138.

Several hitherto imperfectly described diseases of the Tongue, are next minutely diagnosed and their treatment indicated; such as the cracked tongue of the dyspeptic, cured by restoring the condition of the stomach; the ulcers which occur as the sequelae of syphilis, cured by opium and calomel in moderate quantities; soft, elastic, imperfectly defined tumors, slowly and imperfectly suppurating, forming deep sinus's and continuing a long time without affecting the glands of the neck or the health materially, &c. This is often mistaken for cancer, but it is not cancer — it is a curable disease. Regulate the secretions of the digestive organs, liver, &c., if they are defective; but the principal reliance must be placed upon a solution of arsenic in full doses. Sarsaparilla and bichloride of mercury are also occasionally serviceable.

Lectures 17 and 18 treat of Paralysis in its various forms and are succeeded by a lecture on extraction of foreign bodies, in which the lecturer gives full directions to his pupils how to proceed in the various emergencies. If a foreign body is lodged in the upper part of the æsophagus and it cannot be reached, and great difficulty is experienced in the attempts to dislodge it, he advises to let it alone and nature will generally do what is wanted. He has never seen ultimate harm arising from a foreign body stuck in the æsophagus. If it presses upon the trachea and obstructs respiration, and immediate death is threatened, open first the trachea and then search in the æsophagus for the body. The operation of æsophagotomy can scarcely ever be necessary. Sometimes the foreign body, as a pin, for instance, has pricked the throat and passed on and the patient will for one or two days suppose it is still lodged in the passage. But our author is skeptical in relation to those cases in which a large number of pins or needles have been said to have been swallowed, and have finally after long and devious journeyings made their way out at various parts of the body; and equally in relation to those no less marvellous cases in which a whole paper of pins have spontaneously run themselves into the body, as forks and skewers were wont to do in the days of Salem witchcraft.

"It is ridiculous to suppose that a paper of needles could run in of themselves. You know how the girls who are magnetised *deceive* and *cheat*. They pretend to read with the back of their heads, and prophecy all sorts of stuff, and it is just the same here. This woman was humbugging herself in one way as they do in another. I do not advise you however to expose such persons; for that is neither a kind nor a right thing to do. I have before said that some of the very best disposed young women will, when under the influence of hysterical disease, play tricks of this description." p. 180.

An emetic, when a foreign body is lodged in the trachea, is useless and even dangerous. Make an opening in the trachea, and as low as you can generally, proceed slowly, tying the vessels as you progress lest the blood be drawn into the opening when made and the patient die in consequence —separate the parts mostly with a blunt instrument (a knife with a silver blade perhaps). If, when the trachea is opened, the body does not come to the orifice, you are not to irritate the trachea and bronchiae by a long continued and useless probing, but you should try the method by which the Baronet lately saved the life of Mr. Brunel — elevate the body by the heels on an inclined plane, and by a sudden concussion upon the back, the foreign body may be expelled through the glottis. This latter procedure will fail, by inducing a sudden spasm of the glottis, when the foreign body strikes it, unless the trachea is previously laid open. p. 184.

One remark in relation to fistula in ano is worthy of remembrance. Sir Benjamin believes that all anal fistulæ commence from within, and that they have their internal orifice almost invariably just within the sphincter, and he concludes by saying,

"I am now satisfied, and have been for a long time, that the division of a fistula which extends above the inner orifice is quite an unnecessary proceeding." p. 193.

Tumors are the subject of the 23d, 24th, and 25th lectures. In relation to the propriety of operating for scirrous mammæ, a great contrariety of opinion continues to exist. Mr. Cline and Sir Everard Home, seldom would consent to the operation, while others and especially many of the French surgeons operate in nearly all cases. Sir Benjamin finds also but few cases in which he deems the operation warrantable.

"Where the skin is perfectly sound; where the nipple is not retracted; where there is no diseased gland in the axilla; where there is no sign of internal mischief; where there is no adhesion of the breast to the parts below; and where the patient is not very much advanced in life, I should say that there is a reasonable chance of an operation making a cure. I do not intend to say that in all the excepted cases there will be a permanent cure — far from it; but there will be in some instances, and the chance of it may be sufficient to warrant you in recommending the patient to submit to the operation."

We may sometimes, however, operate to give respite and a relief from present sufferings. But if the skin is thoroughly diseased, even this may not be expected. We think we have seen some of the doctors referred to in the following passage:

"When a practitioner tells me that he has been particularly successful in the operation for scirrous tumors of the breast, I am always satisfied that there has been a want of accuracy in the diagnosis. I remember a gentleman stating that he had performed this operation ten times, and that the disease had not returned in a single instance."

The question of the administration of mercury in syphilis, is debated and illustrated at sufficient length. The author's opinion may be gathered from the following summary:

"I am sure that experience proves to me, and it will to you, that we find no remedy having the same power to extinguish venereal disease as mercury, but then it must be not only judiciously administered at the time, but care must be taken that it is only employed in proper cases; it may do great harm if it be improperly used."

Nervous and hysterical diseases follow. In diseases of the hip joint he is sufficiently orthodox.

"In all cases of affection of the hip-joint, without inquiring into the nature of the disease, the first thing that you have to do is to keep the joint in a state of perfect repose."

And it may be for six months, or twelve, or longer, that this plan must be pursued. The concluding chapters treat of diseases of the rectum, antrum maxillare, and of encysted tumors, which together with every page of the book, we recommend to the perusal of all our readers. We pledge them, in these conversational lectures of Sir Benjamin, a substantial repast: and we are happy also to add, that they may, in this volume, see the distinguished Baronet alone, unannoyed by the presence of any obseqious and troublesome valet or umbra, in the livery of an American annotator.

